# Phase Transition in Long-Range Percolation on Bipartite Hierarchical Lattices

**DOI:** 10.1155/2013/172393

**Published:** 2013-10-29

**Authors:** Yilun Shang

**Affiliations:** Singapore University of Technology and Design, 20 Dover Drive, Singapore 138682

## Abstract

We propose a family of bipartite hierarchical lattice of order *N* governed by a pair of parameters *ℓ* and *γ*. We study long-range percolation on the bipartite hierarchical lattice where any edge (running between vertices of unlike bipartition sets) of length *k* is present with probability *p*_*k*_ = 1 − exp(−*αβ* ^−*k*^), independently of all other edges. The parameter *α* is the percolation parameter, while *β* describes the long-range nature of the model. The model exhibits a nontrivial phase transition in the sense that a critical value *α*_*c*_ ∈ (0, *∞*) if and only if *ℓ* ≥ 1, 1 ≤ *γ* ≤ *N* − 1, and *β* ∈ (*N*, *N* ^2^). Moreover, the infinite component is unique when *α* > *α*_*c*_.

## 1. Introduction

For an integer *N* ≥ 2, the hierarchical lattice of order *N* is defined by

(1) ΩN ={x=(x1,x2,…):xi∈{0,1,…,N−1},∑ixi<∞}.

The hierarchical distance *d* on *Ω*_*N*_ is defined by

(2) d(x,y)={0,if  x=y,max⁡{i:xi≠yi},if  x≠y,

which satisfies the strong (non-Archimedean) triangle inequality:

(3) $$\begin{array}{c} d\left(x,y\right)\leq \max\left\{d\left(x,z\right),d\left(z,y\right)\right\}, \end{array}$$

for any **x**, **y**, **z** ∈ *Ω*_*N*_. This means that (*Ω*_*N*_, *d*) is an ultrametric space. Roughly speaking, this corresponds to the leaves of an infinite *N*-ary tree, with metric distance half the graph distance.

Some stochastic models based on hierarchical lattices have been studied. The asymptotic long-range percolation on *Ω*_*N*_ is analyzed in [1] for *N* → *∞*. To the best of our knowledge, this is the first paper devoted to (*Ω*_*N*_, *d*). For different purpose, the works [2–4] study the long-range percolation on *Ω*_*N*_ for fixed *N* by using different connection probabilities. The contact process and perturbation analysis on *Ω*_*N*_ for finite *N* have been studied in [5, 6], respectively. Random walks on hierarchical lattices have been examined in [7, 8].

In this paper, we study percolation on a class of bipartite hierarchical lattices, where edges always run between vertices of unlike type. Bipartite graphs have been studied intensively in the literature (see e.g., [9, 10]) and bipartite structure is popular in many social networks including sexual-contact networks [11] and affiliation networks [12], but we have not seen the setup that we consider here. For two integers *ℓ* ≥ 1 and 1 ≤ *γ* ≤ *N* − 1, consider a partition of *Ω*_*N*_ into two sets:

(4) ΩN1=ΩN1(ℓ,γ)={x=(x1,x2,…)∈ΩN:xℓ∈{0,1,…,γ−1}},ΩN2=ΩN2(ℓ,γ)={x=(x1,x2,…)∈ΩN:xℓ∈{γ,γ+1,…,N−1}}.

Vertices in *Ω*_*N*_^1^ and *Ω*_*N*_^2^ are said to have types 1 and 2, respectively. For each *k* ≥ 1, the probability of connection between two vertices **x** and **y** of unlike type such that *d*(**x**, **y**) = *k* is given by

(5) $$\begin{array}{c} p_{k}=1-\exp\left(-\frac{\alpha }{\beta^{k}}\right), \end{array}$$

where 0 ≤ *α* < *∞* and 0 < *β* < *∞*, all connections being independent. Vertices of the same type cannot be connected with each other, and hence the resulting graph is a class of random bipartite graph.

**Figure 1:**
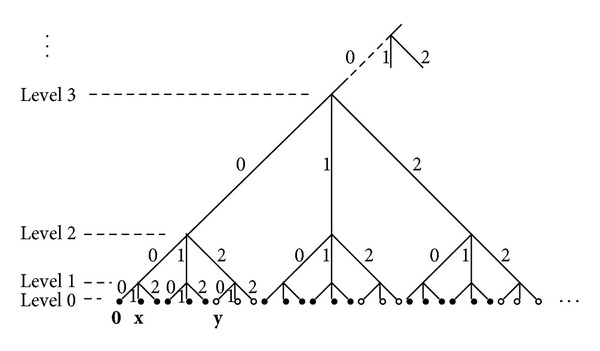
An illustration of bipartite hierarchical lattice (*Ω*_*N*_^1^(*ℓ*, *γ*), *Ω*_*N*_^2^(*ℓ*, *γ*), *d*) of order *N* = 3, *ℓ* = 2, and *γ* = 2. Vertices of type 1 are represented by solid points while those of type 2 hollow points. The distances between three vertices 0 = (0,0, 0,…) ∈ *Ω*_*N*_^1^, **x** = (1,0, 0,…) ∈ *Ω*_*N*_^1^, and **y** = (0,2, 0,…) ∈ *Ω*_*N*_^2^ are *d*(0, **x**) = 1 and *d*(0, **y**) = *d*(**x**, **y**) = 2.

In the above bipartite hierarchical lattice, denoted by (*Ω*_*N*_^1^, *Ω*_*N*_^2^, *d*), vertices of both types are countable and the shortest distance between vertices in *Ω*_*N*_^1^ and *Ω*_*N*_^2^ is *ℓ*. The vertices in (*Ω*_*N*_^1^, *Ω*_*N*_^2^, *d*) can be represented by the leaves at the bottom of an infinite regular tree, where *N* branches emerge from each inner node, see Figure 1. The distance between two vertices (leaves at level 0) is the number of levels from the bottom to their most recent common ancestor. The partition of types for vertices is determined by their ancestors at level *ℓ*; in other words, we need to track back at least *ℓ* levels to find the most recent common ancestor of two vertices of unlike type.

Two vertices **x**, **y** ∈ (*Ω*_*N*_^1^, *Ω*_*N*_^2^, *d*) are in the same component if there exists a finite sequence **x** = **x**_0_, **x**_1_,…, **x**_*n*_ = **y** such that each pair of vertices **x**_*i*−1_ and **x**_*i*_ has different types and shares an edge for *i* = 1,…, *n*. In our model, the parameter *β* > 0 describes the long-range nature, while we think of *α* ≥ 0 as a percolation parameter. We are interested in studying when there is a nontrivial percolation threshold in (*Ω*_*N*_^1^, *Ω*_*N*_^2^, *d*), namely, the critical percolation value *α*_*c*_ ∈ (0, *∞*). Our results for phase transition are analogous to those in the monopartite counterpart (*Ω*_*N*_, *d*) (see [3]). The similar (comparable) behavior of phase transitions in bipartite and corresponding monopartite networks has also been observed in other percolation contexts (see the discussion in Section 2).

The rest of the paper is organized as follows. The results are stated and discussed in Section 2, and the proofs are given in Section 3.

## 2. Results

Let |*S*| be the size of a vertex set *S*. The connected component containing the vertex **x** is denoted by *C*(**x**). By definition, the origin 0 ∈ *Ω*_*N*_^1^(*ℓ*, *γ*) for all *ℓ* ≥ 1 and 1 ≤ *γ* ≤ *N* − 1. Since, for all **x** ∈ *Ω*_*N*_^1^(*ℓ*, *γ*) and **y** ∈ *Ω*_*N*_^2^(*ℓ*, *N* − *γ*), |*C*(**x**)| and |*C*(**y**)| have the same distribution, it suffices to consider only |*C*(0)| without loss of generality. The percolation probability is defined as

(6) $$\begin{array}{c} \theta \left(\ell ,\gamma ,\alpha ,\beta \right)=P\left(\left|C\left(0\right)\right|=\infty \right), \end{array}$$

and the critical percolation value is defined as

(7) $$\begin{array}{c} \alpha_{c}=\alpha_{c}\left(\ell ,\gamma ,\beta \right)=\inf\left\{\alpha \geq 0:\theta \left(\ell ,\gamma ,\alpha ,\beta \right)>0\right\}, \end{array}$$

which is nondecreasing in *β* for any given *ℓ* and *γ*.

**Theorem**

Assume that *ℓ* ≥ 1, 1 ≤ *γ* ≤ *N* − 1 and that 0 < *β* < *∞*. One has the following:

- If *β* ≤ *N*, then *α*_*c*_ = 0.
- If *β* ≥ *N* ^2^, then *α*_*c*_ = *∞*.
- If *N* < *β* < *N* ^2^, then 0 < *α*_*c*_ < *∞*.

Moreover, there is almost surely at most one infinite component when *α* > *α*_*c*_.

**Remark 2**

The critical value *α*_*c*_ = *α*_*c*_(*ℓ*, *γ*, *β*) turns out to be a function of only *β* irrespective of the values of *ℓ* and *γ*. Koval et al. [3] showed the same behavior of *α*_*c*_ for percolation in the monopartite lattice *Ω*_*N*_. This analogy of phase transition has been recognized in other percolation problems in statistical physics. An example is the *AB* percolation introduced by Mai and Halley [13] for the study of gelation processes. In this model, each vertex of an infinite connected graph *G* is assigned one of two states, say *A* and *B*, with probability *p* and 1 − *p*, respectively, independently of all other vertices. Edges with two end-vertices having unlike states (called *AB* bonds) are occupied. Thus, the *AB* percolation can be viewed as a bond percolation with occupation probability 2*p*(1 − *p*) (although some dependence is involved, namely, no odd path of *AB* bonds exists). Appel and Wierman [14] proved that *AB* percolation does not occur for any value of *p* ∈ [0,1] on a bipartite square lattice with bipartition *V*_1_ = {*v* = (*v*_*x*_, *v*_*y*_) ∈ *ℤ* ^2^ : *v*_*x*_ − *v*_*y*_  is  odd} and *V*_2_ = {*v* = (*v*_*x*_, *v*_*y*_) ∈ *ℤ* ^2^ : *v*_*x*_ − *v*_*y*_  is  even} such that *ℤ* ^2^ = *V*_1_ ∪ *V*_2_. In other words, the bond percolation cannot occur on the above bipartite square lattice for occupation probability 2*p*(1 − *p*) ≤ 1/2. This is consistent with the classical result which says that bond percolation on *ℤ* ^2^ does not occur when occupation probability ≤1/2 (see, e.g., [15, 16]). Other comparable *AB* percolation thresholds for monopartite and bipartite high-dimensional lattices can be found in [17].

Another example is the biased percolation [18, 19] on infinite scale-free networks with a power-law degree distribution *P*(*k*) ∝ *k* ^−*γ*^. In this model, an edge between vertices with degrees *k*_1_ and *k*_2_ is occupied with probability proportional to (*k*_1_ *k*_2_)^−*α*^. By using generating function method, Hooyberghs et al. [9] showed that biased percolation on a bipartite scale-free network with two bipartition sets following degree distributions *P* ^*A*^(*k*) ∝ *k* ^−*γ*_*A*_^ and *P* ^*B*^(*k*) ∝ *k* ^−*γ*_*B*_^, respectively, has the same critical behaviors with biased percolation on a monopartite scale-free network when *γ*_*A*_ = *γ*_*B*_ = *γ*.

**Remark**

The uniqueness of the infinite component holds here for the same reason as the uniqueness result for the percolation graph of *Ω*_*N*_ (see [3, Theorem 2]). Note that our graph resulting from (*Ω*_*N*_^1^, *Ω*_*N*_^2^, *d*) can be viewed as a spanning subgraph of that from (*Ω*_*N*_, *d*).

We consider Theorem 1 as an intermediate step towards the study of percolation on bipartite hierarchical lattices. In particular, one may explore the connectivity at the critical regime *β* = *N* ^2^ and the graph distance (chemical distance) between 0 and a vertex **x**. It is also interesting to study the mean field percolation (*N* → *∞*) and compare it with that on *Ω*_*N*_ [1]. Directed percolation [20] and other meaningful colorings on *Ω*_*N*_ (other than the 2-coloring addressed in this paper) are possible.

## 3. Proofs

We start with some notations. Then we prove Theorem 1.

For a vertex **x** ∈ (*Ω*_*N*_^1^, *Ω*_*N*_^2^, *d*), define *B*_*r*_(**x**) as the ball of radius *r* around **x**; that is, *B*_*r*_(**x**) = {**y** : *d*(**x**, **y**) ≤ *r*}. We make the following observations. Firstly, for any vertex **x**, *B*_*r*_(**x**) contains *N* ^*r*^ vertices. In particular, if *r* < *ℓ*, all vertices in the ball have the same type. Secondly, *B*_*r*_(**x**) = *B*_*r*_(**y**) if *d*(**x**, **y**) ≤ *r*. Finally, for any **x**, **y**, and *r*, we have either *B*_*r*_(**x**) = *B*_*r*_(**y**) or *B*_*r*_(**x**)∩*B*_*r*_(**y**) = *∅*.

For a set *S* of vertices, denote by S-=ΩN∖S its complement. Let *C*_*n*_(**x**) be the component of vertices that are connected to **x** by a path using only vertices within *B*_*n*_(**x**). For disjoint sets *S*_1_, *S*_2_⊆*Ω*_*N*_, we denote by *S*_1_↔*S*_2_ the event that at least one edge joins a vertex in *S*_1_ to a vertex in *S*_2_. *S*_1_↮*S*_2_ means the event that such an edge does not exist. By definition, if *S*_1_, *S*_2_⊆*Ω*_*N*_^*i*^ for *i* = 1 or 2, then *S*_1_↮*S*_2_ occurs with probability 1. Let *C*_*n*_^*m*^(**x**) be the largest component in *B*_*n*_(**x**). If there are more than one such components, just take any one of them as *C*_*n*_^*m*^(**x**). It is clear that |*C*_*n*_^*m*^(**x**)| = max⁡_**y**∈*B*_*n*_(**x**)_ | *C*_*n*_(**y**)| [3].

**Proof**

Let *A*_*k*_ be the event that the origin 0 ∈ *Ω*_*N*_^1^ connects by an edge to at least one vertex at distance *k* in *Ω*_*N*_^2^. By construction, for *k* < *ℓ*, *P*(*A*_*k*_) = 0. For *k* = *ℓ*, there are ((*N* − *γ*)/(*N* − 1))(*N* − 1)*N* ^*k*−1^ vertices in *Ω*_*N*_^2^ at distance *k* from 0. Hence,

(8)   P(Aℓ)=1−(1−pℓ)(N−γ)Nk−1=1−exp⁡(−αβℓ(N−γ)Nℓ−1),

by using (5). For *k* > *ℓ*, there are ((*N* − *γ*)/*N*)(*N* − 1)*N* ^*k*−1^ vertices in *Ω*_*N*_^2^ at distance *k* from 0. Similarly, we obtain

(9) $$\begin{array}{c} P\left(A_{k}\right)=1-\exp\left(-\frac{\alpha \left(N-\gamma \right)\left(N-1\right)}{\beta^{k}N}N^{k-1}\right), \end{array}$$

for *k* > *ℓ*.

Since all the events {*A*_*k*_}_*k*≥1_ are independent and

(10) ∑k=1∞P(Ak)≈1−exp⁡(−αβℓ(N−γ)Nℓ−1)  +α(N−γ)N∑k=ℓ+1∞(Nβ)k

diverges for any 0 < *β* ≤ *N*, 1 ≤ *γ* ≤ *N* − 1, and *α* > 0, infinitely many of *A*_*k*_ occur with probability 1 by the second Borel-Cantelli lemma. Thus, *θ*(*ℓ*, *γ*, *α*, *β*) = 1 for any *ℓ* > 1, 1 ≤ *γ* ≤ *N* − 1, *α* > 0, and 0 < *β* ≤ *N*. The result then follows.

**Proof**

We only need to show *α*_*c*_(*ℓ*, *γ*, *N* ^2^) = *∞* by virtue of the monotonicity. Note that, for *j* ≥ *ℓ*, there are (*γ*/*N*)*N* ^*j*^ vertices in *B*_*j*_(0) ∩ *Ω*_*N*_^1^ and ((*N* − *γ*)/*N*)*N* ^*j*^ vertices in *B*_*j*_(0) ∩ *Ω*_*N*_^2^. Hence, by the comments in the proof of (i) and taking *β* = *N* ^2^, for any *j* ≥ *ℓ*, we obtain

(11) P(Bj(0)⟷Bj(0)¯) =1−(∏k=j+1∞(1−pk)((N−γ)/N)(N−1)Nk−1)(γ/N)Nj  ·(∏k=j+1∞(1−pk)(γ/N)(N−1)Nk−1)((N−γ)/N)Nj =1−exp⁡(−2α(N−γ)(N−1)γNjN4×∑k=1∞Nk+j−1N2(k+j−1)) =1−exp⁡(−2α(N−γ)γN3),

which is strictly less than 1 for any finite *α* ≥ 0.

Let *n*_*ℓ*_ = 0 and $n_{i+1}=\inf\{n\geq n_{i}:B_{n_{i}}(0)↮\bar{B_{n}(0)}\}$. We have

(12) $$\begin{array}{c} \left\{\left|C\left(0\right)\right|=\infty \right\}\subseteq \overset{\infty }{\underset{i=\ell }{⋂}}\left\{B_{n_{i}}\left(0\right)⟷\bar{B_{n_{i}}\left(0\right)}\right\}. \end{array}$$

Since the events $\left\{B_{n_{i}}(0)↔\bar{B_{n_{i}}(0)}\right\}$ are independent and all have the same probability strictly less than 1,

(13) $$\begin{array}{c} P\left(B_{n_{i}}\left(0\right)⟷\bar{B_{n_{i}}\left(0\right)},\text{for}\;\text{any}\;i\geq \ell \right)=0. \end{array}$$

Consequently, there exists an *i* such that $B_{n_{i}}(0)↮\bar{B_{n_{i}}(0)}$ with probability 1. It follows from (12) that *θ*(*ℓ*, *γ*, *α*, *N* ^2^) = 0 for all *α* ≥ 0. This implies *α*_*c*_(*ℓ*, *γ*, *N* ^2^) = *∞*.

**Proof**

The positivity of *α*_*c*_ is a direct consequence of the proof of Theorem 1(b) in [3]. Since the percolation graph of (*Ω*_*N*_^1^, *Ω*_*N*_^2^, *d*) can be viewed as a spanning subgraph of that of (*Ω*_*N*_, *d*), the percolation cluster *C*(0) is almost surely finite; namely, *θ*(*ℓ*, *γ*, *α*, *β*) = 0, for *α* small enough.

Now we turn to the proof of finiteness of *α*_*c*_. The main technique to be used is an iteration involving the tail probability of binomial distributions [3, 21]. Since *β* < *N* ^2^, we choose an integer *K* and a real number *δ* such that

(14) $$\begin{array}{c} \sqrt{\beta }<\delta \leq {\left(N^{K}-1\right)}^{1/K}. \end{array}$$

Clearly, 1 < *δ* < *N*. For *n* ≥ 1, let

(15) an=P(|CnKm(0)∩ΩN1|≥γNδnK,|CnKm(0)∩ΩN2|≥N−γNδnK),

and analogously,

(16) bn=P(|CnK(0)∩ΩN1|≥γNδnK,|CnK(0)∩ΩN2|≥N−γNδnK).

Here, *a*_*n*_ is the probability that the largest component of a ball of radius *nK* contains at least (*γ*/*N*)*δ* ^*nK*^ vertices in *Ω*_*N*_^1^ and at least ((*N* − *γ*)/*N*)*δ* ^*nK*^ vertices in *Ω*_*N*_^2^. Such a ball is said to be *good*. We set *a*_0_ = *b*_0_ = 1 by convention. It is clear that, for *α* > 0, all *a*_*n*_ and *b*_*n*_ are positive, since *nK* is a finite number and the connection probability in (5) is positive.

In what follows, we will prove *α*_*c*_ < *∞* in two steps.

**Step 1**

We show that there exists some *α* > 0 such that *a*_*n*_ converges to 1 exponentially fast; namely, 1 − *a*_*n*_ ≤ exp⁡(−*cn*) for some *c* > 0.

**Step 2**

We show that there exists some *α* > 0 such that liminf⁡_*n*→*∞*_ *b*_*n*_ > 0.

We start with Step 1. To this end, denote by *ℕ* the nonnegative integers. We can naturally label the vertices in *Ω*_*N*_ via the map *f* : *Ω*_*N*_ → *ℕ* as

(17) $$\begin{array}{c} f:x=\left(x_{1},x_{2},\ldots \right)\mapsto \overset{\infty }{\underset{i=1}{\sum }}x_{i}N^{i-1}. \end{array}$$

This order agrees with the depiction in Figure 1. A ball of radius *nK* is said to be *very good* if it is good and its largest component connects by an edge to the largest component of the first (as per the aforementioned order) good subball in the same ball of radius (*n* + 1)*K*. Clearly, the first good subball of radius *nK* in a ball of radius (*n* + 1)*K* is very good. Condition (14) implies that (*N* ^*K*^ − 1)*δ* ^*nK*^ ≥ *δ* ^(*n*+1)*K*^. Thus we assert that the ball *B*_(*n*+1)*K*_(0) is good if (a) it contains *N* ^*K*^ − 1 good subballs of radius *nK*, and (b) all these good subballs are very good.

The number of good subballs of radius *nK* in a ball of radius (*n* + 1)*K* has a binomial distribution Bin(*N* ^*K*^, *a*_*n*_) with parameters *N* ^*K*^ and *a*_*n*_. Given the collection of good subballs, the probability that the first such good subball is very good equals to 1. Fix any of the other good subballs, say *B*; the probability that *B* is not very good is upper bounded by

(18) (1−p(n+1)K)ad+bc≤(1−p(n+1)K)(2γ(N−γ)/N2)  δ2nK=exp⁡(−2αγ(N−γ)βKN2(δ2β)nK)≔εn,

where *a* and *b* are the number of vertices in the largest component of the first good subball in *Ω*_*N*_^1^ and *Ω*_*N*_^2^, respectively; likewise, *c* and *d* are the number of vertices in the largest component of the subball *B* in *Ω*_*N*_^1^ and *Ω*_*N*_^2^, respectively. By definition, we have *a*, *c* ≥ (*γ*/*N*)*δ* ^*nK*^, *b*, *d* ≥ ((*N* − *γ*)/*N*)*δ* ^*nK*^, and the distance between two vertices in a ball of radius (*n* + 1)*K* is at most (*n* + 1)*K*.

Therefore, the probability for any of the other good subballs *B* to be very good is at leat 1 − *ε*_*n*_. Thus, the number of very good subballs is stochastically larger than a random variable obeying a binomial distribution Bin(*N* ^*K*^, *a*_*n*_(1 − *ε*_*n*_)). From the above comments (a) and (b) and the definition of *a*_*n*_, it follows that

(19) $$\begin{array}{c} a_{n+1}\geq P\left(\text{Bin}\left(N^{K},a_{n}\left(1-\varepsilon_{n}\right)\right)\geq N^{K}-1\right). \end{array}$$

In general, we have the following inequality for the tail of binomial random variable:

(20) $$\begin{array}{c} P\left(\text{Bin}\left(n,p\right)\geq n-1\right)\geq 1-\left(\begin{array}{c} n\\ 2 \end{array}\right){\left(1-p\right)}^{2}. \end{array}$$

By (19), (20), and writing *ξ*_*n*_ = 1 − *a*_*n*_, we obtain

(21) ξn+1=1−an+1≤(NK2)(1−an+anεn)2≤(NK2)(1−an+εn)2=(NK2)(ξn+εn)2.

We can choose *c* > 0 large enough so that $4\left(\begin{array}{c} N^{K}\\ 2 \end{array}\right)\leq \exp(c)$, and then we choose *α* large enough so that (c) *ε*_*n*_ ≤ exp⁡(−*c*(*n* + 1)) and (d) *ξ*_1_ ≤ exp⁡(−2*c*) hold. To see (c), note that *β* < *δ* ^2^ and then

(22) εn=exp⁡(−2αγ(N−γ)βKN2(δ2β)nK)≤((βδ2)2Kαγ(N−γ)/βkN2)n.

To see (d), note that lim⁡_*α*→*∞*_ *ε*_0_ = 0, *ξ*_0_ = 0 and by (21) we obtain

(23) $$\begin{array}{c} \xi_{1}=1-a_{1}\leq \left(\begin{array}{c} N^{K}\\ 2 \end{array}\right){\left(\xi_{0}+\varepsilon_{0}\right)}^{2}, \end{array}$$

which also approaches 0.

According to our above choice of *c* and *α*, we have inductively, if *ξ*_*n*_ ≤ exp⁡(−*c*(*n* + 1)), then

(24) ξn+1≤(NK2)(ξn+εn)2≤4(NK2)exp⁡(−2c(n+1))≤exp⁡(−c(2n+1))≤exp⁡(−c(n+2)),

which implies that *ξ*_*n*_ ≤ exp⁡(−*c*(*n* + 1)) ≤ exp⁡(−*cn*) for all *n* ∈ *ℕ*. We then finish the proof of Step 1.

For Step 2, recalling the definition of *b*_*n*_, we claim that

(25) $$\begin{array}{c} b_{n+1}\geq b_{n}\cdot P\left(\text{Bin}\left(N^{K}-1,a_{n}\left(1-\varepsilon_{n}\right)\right)\geq N^{K}-2\right). \end{array}$$

In deed, if |*C*_*nK*_(0)∩*Ω*_*N*_^1^ | ≥(*γ*/*N*)*δ* ^*nK*^ and |*C*_*nK*_(0)∩*Ω*_*N*_^2^ | ≥((*N* − *γ*)/*N*)*δ* ^*nK*^, then *B*_*nK*_(0) is the first good subball in the derivation above. If this component is connected to at least *N* ^*K*^ − 2 other large components in *B*_(*n*+1)*K*_(0) as above, then the component containing the origin in *B*_(*n*+1)*K*_(0) has (*γ*/*N*)*δ* ^*nK*^(*N* ^*K*^ − 1)≥(*γ*/*N*)*δ* ^(*n*+1)*K*^ vertices in *Ω*_*N*_^1^ and ((*N* − *γ*)/*N*)*δ* ^*nK*^(*N* ^*K*^ − 1)≥((*N* − *γ*)/*N*)*δ* ^(*n*+1)*K*^ vertices in *Ω*_*N*_^2^. Thus, the inequality (25) follows.

A simple coupling gives

(26) P(Bin(NK−1,an(1−εn))≥NK−2)  ≥P(Bin(NK,an(1−εn))≥NK−1).

Hence, we derive that the right-hand side of (26) converges to 1 exponentially fast by exploiting (20) and the fact that *a*_*n*_(1 − *ε*_*n*_) converges to 1 exponentially fast. It then follows from (25) that

(27) $$\begin{array}{c} b_{n+1}\geq b_{n}\left(1-\exp\left(-cn\right)\right), \end{array}$$

for some *c* > 0. Hence,

(28) $$\begin{array}{c} b_{n+1}\geq b_{1}\overset{n}{\underset{k=1}{\prod }}\left(1-\exp\left(-ck\right)\right). \end{array}$$

It is direct to check that

(29) ln⁡⁡(∏k=1n(1−exp⁡(−ck)))≥ln⁡(1−∑k=1nexp⁡⁡(−ck))≥ln⁡(1−2exp⁡(−c)1−exp⁡(−c))>−∞,

and hence ∏_*k*=1_^*n*^(1 − exp⁡(−*ck*)) > 0 for all *n*. Since *b*_1_ > 0, inequality (28) yields

(30) $$\begin{array}{c} \underset{n\to \infty }{\mathrm{liminf}}\;b_{n}>0, \end{array}$$

as desired.
